# Supplementary material for: Performance of Genotype MTBDRsl V2.0 over the Genotype MTBDRsl V1 for detection of second line drug resistance: An Indian perspective
Source: PLoS One. 2020 Mar 4;15(3):e0229419. doi: 10.1371/journal.pone.0229419 (PMC7055869; doi:10.1371/journal.pone.0229419)
Supplement: S1 Data — (PDF) [file pone.0229419.s001.pdf]

S.NO	Sample Lab ID	Sample	AFB	State	BACTEC MGIT SIRE RESULTS			
					S	I	R	E
1	3657/08	SP	Neg	Delhi	S	R	R	R
2	10064/12	SP	1+	UP	R	R	R	S
3	10134/12	CSF	Neg	UP	R	R	R	S
4	12801/14	PUS	Neg	Delhi	R	R	R	R
5	12880/15	SP	3+	Delhi	S	R	R	S
6	14098/15	BAL	Scanty	Bihar	R	R	R	R
7	14164/15	LNA	Neg	Delhi	R	R	R	R
8	9033/12	PF	Neg	UP	S	R	R	R
9	10017/12	CSF	Neg	delhi	R	R	R	S
10	10024/12	CSF	Neg	Ghaziabad	S	R	R	S
11	10259/13	GA	neg	Delhi	S	R	R	S
12	10489/13	SP	Neg	Haryana	S	R	R	S
13	10951/13	SP	Neg	Delhi	S	R	R	S
14	10963/13	SP	Neg	Delhi	R	R	R	S
15	10966/13	CSF	Neg	Bihar	R	R	R	S
16	11489/13	SP	Neg	Mizorm	R	R	R	S
17	L-330	SP	1+	Mohali	R	R	R	R
18	12944/15	PF	Neg	Delhi	S	R	R	S
19	12975/15	PF	Neg	Delhi	S	R	R	S
20	L-788	SP	Scanty	Jalandhar	R	R	R	S
21	13051/15	SP	Neg	Delhi	R	R	R	S
22	TB-13272 NE	SP	1+	Northeast	R	R	R	S
23	13457/15	BAL	Neg	New Delhi	S	R	R	S
24	13574/15	CSF	Neg	Delhi	R	R	R	R
25	13789/15	SP	2+	New Delhi	S	R	R	S
26	13797/15	BAL	Neg	Delhi	S	R	R	S
27	13841/15	SYF	Neg	Delhi	R	R	R	S
28	TB-14068-NE	SP	1+	Northeast	R	R	R	R
29	TB-14336 NE	SP	1+	Northeast	R	R	R	R
30	14086/15	SP	Neg	Delhi	R	R	R	R
31	TB-14351NE	SP	1+	Delhi	R	R	R	R
32	TB-10543	Pleural fluid	Neg	Haryana	R	R	R	S
33	14290/15	Bx	Neg	Delhi	S	R	R	S
34	14463/15	SP	3+	New Delhi	S	R	R	S
35	4184/08	Urine	Neg	Delhi	S	R	R	R
36	5084/09	SP	Neg	Delhi	S	R	R	S
37	9020/12	lna	Neg	Delhi	R	R	R	R
38	L-121	SP	1+	Punjab	R	R	R	S
39	L-148	SP	1+	Punjab	S	R	R	R
40	L-178	SP	2+	Ludhiana	R	R	R	R
41	L-31	SP	3+	Jalandhar	S	R	R	R
42	12062/13	PUS	Neg	Delhi	R	R	R	R
43	L-350	SP	3+	Sangrur	S	R	R	R
44	L-371	SP	2+	Amritsar	S	R	R	R
45	L-442	SP	1+	Amritsar	R	R	R	S
46	13182/15	CSF	Neg	New Delhi	R	R	R	R
47	13577/15	Pus	Scanty	New Delhi	R	R	R	R
48	13665/15	SP	2+	Delhi	R	R	R	R
49	13762/15	LNA	Scanty	New Delhi	R	R	R	R
50	13871/15	SP	1+	UP	R	R	R	R

51	TB-13291-NE	SP	1+	Northeast	R	R	R	R
52	TB-13691 NE	SP	1+	Northeast	R	R	R	S
53	TB-14341NE	SP	1+	Northeast	R	R	R	R
54	TB-14344NE	SP	Scanty	Northeast	R	R	R	S
55	TB-14348NE	SP	Scanty	Northeast	R	R	R	R
56	TB-14349NE	SP	Scanty	Northeast	R	R	R	S
57	TB-14352 NE	SP	Scanty	Northeast	R	R	R	R
58	TB-14338NE	SP	1+	Northeast	R	R	R	R
59	TB-14354 NE	SP	1+	Northeast	S	R	R	S
60	L-545	SP	Scanty	Punjab	R	R	R	R
61	TB-14342 NE	SP	1+	Northeast	R	R	R	R
62	TB-13167	CSF	Neg	Delhi	R	R	R	S
63	TB-14133	CSF	nEG	DELHI	S	R	R	S
64	TB-11378	SP	2+	Delhi	R	R	R	R
65	TB-12679	csf	nEG	UP	S	R	R	S
66	L-287	Sputum	1+	Punjab	R	R	R	R
67	TB-10216	Sputum	3+	UP	S	R	R	S
68	TB-12690	Sputum	Neg	Delhi	S	R	R	S
69	TB-14345 NE	SP	2+	Punjab	S	R	R	R
70	L-141	sPUTUM	2+	Punjab	S	R	R	S
71	L-744	Sputum	Scanty	Jalandhar	R	R	R	S
72	TB-2016	Sputum	2+	DELHI	R	R	R	R
73	TB-14347NE	SP	Scanty	Northeast	R	R	R	R
74	TB-14350NE	SP	Scanty	Northeast	R	R	R	R
75	TB-14353 NE	SP	1+	Northeast	R	R	R	R
76	L-784	SP	2+	Punjab	S	R	R	S
77	TB-14864	Sputum		Northeast	S	R	R	S
78	TB-14846	Sputum		Northeast	S	R	R	R
79	TB-14708	Sputum		Northeast	S	R	R	S
80	TB-14721	Sputum		Northeast	R	R	R	S
81	TB-14720	sputum		Northeast	R	R	R	R
82	TB-14827	sputum		Northeast	S	R	R	S
83	TB-13577	Pus		Northeast	R	R	R	S
84	TB-15035	SPUTUM		Northeast	R	R	R	S
85	TB-14935	sputum		Northeast	R	R	R	S
86	TB-10489 New	Sputum		Northeast	R	R	R	S
87	TB-15168	sputum		Northeast	R	R	R	R
88	11716/13	PUS	Scanty	Sikkim	R	R	R	R
89	12529/14	CSF	Neg	New Delhi	S	R	R	S
90	12633/14	PF	Neg	Delhi	R	R	R	S
91	12766/14	SP	Neg	Delhi	S	R	R	S
92	L-725	SP	2+	Jalandhar	R	R	R	S
93	12968/15	CSF	neg	Delhi	S	R	R	S
94	13000/15	GA	Neg	Delhi	S	R	R	S
95	13040/15	SP	Neg	Delhi	R	R	R	S
96	13245/15	SP	3+	Delhi	R	R	R	S
97	TB-14144	SP		Delhi	S	R	R	R
98	TB-14156	SP		Delhi	S	R	R	S
99	Tb-14292	SP		Delhi	R	R	R	S
100	TB-14141	SP		Delhi	S	S	R	R
101	11716/13	PUS	Scanty	Sikkim	R	R	R	R
102	12529/14	CSF	Neg	New Delhi	S	R	R	S

103	12633/14	PF	Neg	Delhi	R	R	R	S
104	12766/14	SP	Neg	Delhi	S	R	R	S
105	L-725	SP	2+	Jalandhar	R	R	R	S
106	12968/15	CSF	neg	Delhi	S	R	R	S
107	13000/15	GA	Neg	Delhi	S	R	R	S
108	13040/15	SP	Neg	Delhi	R	R	R	S
109	13245/15	SP	3+	Delhi	R	R	R	S
110	TB-14144	SP		Delhi	S	R	R	R
111	TB-14156	SP		Delhi	R	R	R	S
112	Tb-14292	SP		Delhi	S	R	R	S
113	TB-14141	SP		Delhi	S	R	R	S

BACTEC MGIT SECOND LINE RESULTS					LP		
**O**	**K**	**A**	**C**	**REMARKS**	**gyrA WT**	**gyrA MT**	**rrs WT**
S	S	S	S	SENSITIVE	+	-	+
R	S	S	S	ofloxacin	+	m1	+
R	S	S	S	ofloxacin	WT3	m3c	+
R	S	S	S	ofloxacin	WT3	m3c	+
R	S	S	S	ofloxacin	+	m3c	+
R	S	S	S	ofloxacin	WT2	m1	+
R	S	S	S	ofloxacin	WT2	m1	+
S	S	S	S	SENSITIVE	+	-	+
R	S	S	S	ofloxacin	+	-	+
S	S	S	S	SENSITIVE	+	-	+
S	S	S	S	SENSITIVE	+	-	+
S	S	S	S	SENSITIVE	+	-	+
S	S	S	S	SENSITIVE	+	-	+
S	S	S	S	SENSITIVE	+	-	+
R	R	R	S	OKA	WT2	m1	WT1
S	S	S	S	SENSITIVE	+	-	+
R	S	S	S	ofloxacin	+	m1	+
S	S	S	S	SENSITIVE	+	-	+
S	S	S	S	SENSITIVE	+	-	+
R	S	S	S	ofloxacin	WT3	m3c	+
s	S	S	S	SENSITIVE	+	-	+
R	S	S	S	ofloxacin	WT3	m3c	+
S	S	S	S	SENSITIVE	+	-	+
S	S	S	S	SENSITIVE	+	-	+
S	S	S	S	SENSITIVE	+	-	+
S	S	S	S	SENSITIVE	+	-	+
S	S	S	S	SENSITIVE	+	-	+
R	S	S	S	ofloxacin	+	m1	+
R	S	S	S	ofloxacin	WT3	Mut3D	+
S	S	S	S	SENSITIVE	+	-	+
R	S	S	S	ofloxacin	WT3	m3c	+
R	S	S	S	ofloxacin	WT3	Mut3B,3C,3D	+
S	S	S	S	SENSITIVE	+	-	+
R	R	R	S	OKA	+	Mut1,Mut3c	+
R	S	S	S	ofloxacin	WT3	m3c	+
R	S	S	S	ofloxacin	WT3	m3c	+
R	S	S	S	ofloxacin	WT3	mut3a	+
S	S	S	S	SENSITIVE	+	-	+
S	S	S	S	ofloxacin	WT3	m3c	+
S	S	S	S	SENSITIVE	+	-	+
R	R	R	R	OKAC	WT3	m3c	WT1
R	S	S	S	ofloxacin	WT3	m3c	+
S	S	S	S	SENSITIVE	+	-	+
S	S	S	S	SENSITIVE	+	-	+
S	S	S	S	SENSITIVE	+	-	+
R	S	S	S	ofloxacin	WT2	m1	+
R	S	S	S	ofloxacin	WT3	mut3a	+
R	S	S	S	ofloxacin	WT3	m3c	+
R	S	S	S	ofloxacin	+	Mut1,Mut3c	+
R	S	S	S	ofloxacin	+	muta3a,mut3c	+

S	S	S	S	SENSITIVE	+	-	+
S	S	S	S	SENSITIVE	+	-	+
R	R	S	S	OK	WT3	m3c	+
S	S	S	S	SENSITIVE	+	-	+
S	S	S	S	SENSITIVE	+	-	+
S	S	S	S	SENSITIVE	+	-	+
R	R	R	R	OKAC	WT3	m3c	WT1
R	S	S	S	ofloxacin	WT3	m3c	+
S	S	S	S	SENSITIVE	+	-	+
R	S	S	S	ofloxacin	+	-	+
R	S	S	S	ofloxacin	WT3	m3c	+
S	S	S	S	SENSITIVE	+	-	+
S	S	S	S	SENSITIVE	+	-	+
S	S	S	S	SENSITIVE	+	-	+
S	S	S	S	SENSITIVE	+	-	+
R	R	R	R	OKAC	WT3	mut3a	WT1
S	S	S	S	SENSITIVE	+	-	+
S	S	S	S	SENSITIVE	+	-	+
R	S	S	S	ofloxacin	+	m1	+
S	S	S	S	SENSITIVE	+	-	+
S	S	S	S	SENSITIVE	+	-	+
S	S	S	S	ofloxacin	WT3	m3c	+
R	S	S	S	ofloxacin	WT2	m1	+
R	S	S	S	ofloxacin	WT3	Mut3B	+
R	S	S	S	ofloxacin	WT3	MUT3B	+
R	S	S	S	ofloxacin	WT3	m3c	+
S	S	S	S	Sensitive	+	-	+
S	S	S	S	SENSITIVE	+	-	+
S	S	S	S	SENSITIVE	+	-	+
S	S	S	S	Sensitive	+	-	+
S	S	S	S	Sensitive	+	-	+
S	S	S	S	Sensitive	+	-	+
S	S	S	S	ofloxacin	+	Mut2	+
S	S	S	S	SENSITIVE	+	-	+
R	S	S	S	ofloxacin	wt2	m1	+
S	S	S	S	Sensitive	+	-	+
R	S	S	S	ofloxacin	wt2	m1	+
S	S	S	S	SENSITIVE	+	-	+
S	S	S	S	SENSITIVE	+	-	+
S	S	S	S	SENSITIVE	+	-	+
S	S	S	S	SENSITIVE	+	-	+
R	S	S	S	ofloxacin	WT2	m1	+
S	S	S	S	SENSITIVE	+	-	+
S	S	S	S	SENSITIVE	+	-	+
S	S	S	S	SENSITIVE	+	-	+
S	S	S	S	SENSITIVE	+	-	+
S	S	S	S	SENSITIVE	+	-	+
R	S	S	S	ofloxacin	+	m1, m3c	+
S	S	S	S	SENSITIVE	+	-	+
S	S	S	S	SENSITIVE	+	-	+
S	S	S	S	SENSITIVE	+	-	+
S	S	S	S	SENSITIVE	+	-	+

S	S	S	S	SENSITIVE	+	-	+
S	S	S	S	SENSITIVE	+	-	+
R	S	S	S	ofloxacin	WT2	m1	+
S	S	S	S	SENSITIVE	+	-	+
S	S	S	S	SENSITIVE	+	-	+
S	S	S	S	SENSITIVE	+	-	+
S	S	S	S	SENSITIVE	+	-	+
S	S	S	S	SENSITIVE	+	-	+
R	S	S	S	ofloxacin	+	m3c	+
S	S	S	S	SENSITIVE	+	-	+
S	S	S	S	SENSITIVE	+	-	+

A V1.0 Results						
rrs MT	emb WT	emb MT	Remarks	gyrA WT	gyrA MT	gyrb WT
-	wt1	m1b	E	+	-	+
-	+	-	F	+	m1	+
-	+	-	F	WT3	m3C	+
-	+	-	F	WT3	Mut3C	+
-	+	-	F	+	m3c	+
-	+	-	F	Wt2	M1	+
-	+	-	F	Wt2	M1	+
-	+	Mut1B	E	+	-	+
-	wt1	m1a	E	WT2	m1	+
-	+	-	S	+	-	+
-	+	-	S	+	-	+
-	+	-	S	+	-	+
-	+	-	Sen	+	-	+
-	+	-	S	WT3	m3	+
m1	+	-	FI	WT2	m1	+
-	+	-	S	+	-	+
-	+	-	F	+	m1	+
-	+	-	Sen	+	-	+
-	+	-	Sen	+	-	+
-	+	-	F	WT3	m3C	+
-	+	-	Sen	+	-	+
-	+	-	F	WT3	Mut3C	+
-	+	-	Sen	+	-	+
-	+	-	Sen	+	-	+
-	+	-	Sen	+	-	+
-	wt1	m1b	E	+	-	+
-	+	-	Sen	+	-	+
-	+	-	F	+	m1	+
-	+	-	F	Wt3		+
-	+	-	Sen	+	-	+
-	+	-	F	WT3	m3C	+
-	+	-	F	WT3	Mut3B,3C,3D	+
-	+	-	Sen	+	-	+
MUT1	+	-	FI	+	Mut1,Mut3c	+
-	wt1	m1b	FE	WT3	m3C	+
-	wt1	m1b	FE	WT3	Mut3C	+
-	wt1	m1b	FE	Wt3	Mut3a	+
-	+	-	S	+	-	+
-	wt1	m1b	FE	WT3	Mut3C	+
-	+	-	Sen	+	-	+
m1	+	-	FI	WT3	m3c	+
-	wt1	m1b	FE	WT3	m3C	+
-	+	-	S	+	-	+
-	wt1	-	E	+	-	+
-	+	-	S	+	-	+
-	wt1	-	FE	Wt2	M1	+
-	wt1	m1b	FE	Wt3	Mut3a	+
-	wt1	m1a	FE	WT3	m3C	+
-	+	MUT1A	FE	+	Mut1,Mut3c	+
-	wt1	m1b	FE	+	mut1	+

-	wt1	m1b	E	+	-	+
-	+	-	Sen	+	-	+
-	wt1	-	FE	WT3	Mut3c	+
-	+	-	Sen	+	-	+
-	+	-	Sen	+	-	+
-	+	-	Sen	+	-	+
m1	wt1	m1b	FIE	WT3	m3c	+
-	wt1	m1b	FE	WT3	m3C	+
-	+	-	Sen	+	-	+
-	+	-	Sen	+	-	+
-	wt1	m1b	FE	WT3	m3C	+
-	+	-	Sen	+	-	+
-	+	-	Sen	+	-	+
-	+	-	Sen	+	-	+
-	+	-	Sen	+	-	+
-	+	-	FI	WT3	mut3a	WT1
-	+		Sen	+	-	+
-	+	-	S	+	-	+
-	wt1	m1b	FE	+	m1	+
-	+	-	Sen	+	-	+
-	wt1	m1b	E	+	-	+
-	wt1	m1a	FE	WT3	m3C	+
-	wt1	m1a	FE	Wt2	M1	+
-	wt1	m1b	FE	wt3	mut3b	+
-	wt1	m1b	FE	WT3	mut3b	+
-	wt1	m1b	FE	WT3	m3C	+
-	+	-	Sen	+	-	+
-	+	-	Sen	+	-	+
-	+	-	Sen	+	-	+
-	wt1	m1b	E	+	-	+
-	+	-	Sen	+	-	+
-	+	-	Sen	+	-	+
-	wt1	m1b	F	+	Mut2	+
-	+	-	sen	+	-	+
-	+	-	F	Wt2	M1	+
-	+	-	Sen	+	-	+
-	+	-	F	Wt2	M1	+
-	wt1	m1b	E	+	-	+
-	+	-	Sen	+	-	+
-	+	-	Sen	+	-	+
-	+	-	Sen	+	-	+
-	+	-	F	Wt2	M1	+
-	+	-	Sen	+	-	+
-	+	-	Sen	+	-	+
-	+	-	Sen	+	-	+
-	+	-	Sen	+	-	+
-	+	-	Sen	+	-	+
-	+	-	F	+	Mut1,Mut3c	+
-	+	-	Sen	+	-	+
-	+	-	Sen	+	-	+
-	wt1	m1b	E	+	-	+
-	+	-	Sen	+	-	+

-	+	-	Sen	+	-	+
-	+	-	Sen	+	-	+
-	+	-	F	Wt2	M1	+
-	+	-	Sen	+	-	+
-	+	-	Sen	+	-	+
-	+	-	Sen	+	-	+
-	+	-	Sen	+	-	+
-	+	-	Sen	+	-	+
-	+	-	F	+	m3c	Wt1
-	+	-	Sen	+	-	+
-	+	-	Sen	+	-	+

LPA V2.0 Results

gyrb MT	rrs WT	rrs MT	eis WT	EIS MT	Remarks
-	+	-	+	-	SEN
-	+	-	+	-	F
-	+	-	+	-	F
Mut1	+	-	+	-	F
Mut1	+	-	+	-	F
-	+	-	+	-	F
-	+	-	+	-	F
-	+	-	+	-	SEN
Mut1	+	-	+	-	F
-	+	-	S		Sen
-	+	-	+	-	SEN
-	+	-	+	-	SEN
-	+	-	+	-	SEN
-	+	-	+	-	SEN
Mut1	+	m1	+	-	FI
-	+	-	+	-	SEN
-	+	-	+	-	F
-	+	-	+	-	SEN
-	+	-	+	-	SEN
-	+	-	+	-	F
-	+	-	+	-	SEN
Mut1	+	-	+	-	F
-	+	-	+	-	sen
-	+	-	+	-	sen
-	+	-	+	-	SEN
-	+	-	+	-	SEN
-	+	-	+	-	Sen
-	+	-	+	-	F
Mut1	+	-			F
-	+	-	+	-	SEN
-	+	-	+	-	F
mut1	+	-	+	-	F
-	+	-	+	-	SEN
-	+	Mut1	+	-	FI
-	+	-	+	-	F
Mut1	+	-	+	-	F
Mut1	+	-	-	+	F
-	+	-	+	-	SEN
Mut1	+	-	+	-	F
-	+	-	+	-	SEN
-	WT1	m1	+	-	FI
-	+	-	+	-	F
-	+	-	+	-	SEN
-	+	-	+	-	Sen
-	+	-	+	-	SEN
-	+	-	+	-	F
-	+	-	+	-	F
-	+	-	+	-	F
-	+	-	+	-	F
-	+	-	+	-	F

-	+	-	+	-	SEN
-	+	-	+	-	SEN
-	+	-	+	mut1	FK
-	+	-	+	-	SEN
-	+	-	+	-	SEN
-	+	-	+	-	SEN
-	WT1	m1	+	-	FI
-	+	-	+	-	F
-	+	-	Delhi	-	SEN
Mut1	+	-	+	-	F
-	+	-	+	-	F
-	+	-	+	-	Sen
-	+	-	+	-	SEN
-	+	-	+	-	SEN
-	+	-	Sen		Sen
-	Wt1	Mut1	+	-	FI
-	+	-	+	-	SEN
-	+	-	S		sen
-	+	-	+	-	F
-	+	-	Sen		SEN
-	+	-	+	_	SEN
-	+	-	+	-	F
-	+	-	+	-	F
-	+	-	+	-	F
Mut1	+	-	+	-	F
-	+	-	+	-	F
-	+	-	+	-	SEN
-	+	-	+	-	SEN
-	+	-	+	-	SEN
-	+	-	+	-	SEN
-	+	-	+	-	SEN
-	+	-	+		SEN
Mut1	WT1	m1	+	Mut1	FIK
-	+	-	+	-	SEN
-	+	-	+	-	F
-	+	-	+	-	SEN
-	+	-	+	-	F
-	+	-	+	-	SEN
-	+	-	+	-	SEN
-	+	-	+	-	SEN
-	+	-	+	-	SEN
-	+	-	+	-	F
-	+	-		+	SEN
-	+	-		+	SEN
-	+	-		+	SEN
-	+	-		+	SEN
-	+	-	+	-	SEN
-	+	-	+	-	F
-	+	-	+	-	SEN
-	+	-	+	-	SEN
-	+	-	+	-	SEN
-	+	-	+	-	SEN

-	+	-	+	-	SEN
-	+	-	+	-	SEN
-	+	-	+	-	F
-	+	-		+	SEN
-	+	-		+	SEN
-	+	-		+	SEN
-	+	-		+	SEN
-	+	-	+	-	SEN
Mut1	+	-	+	-	F
-	+	-	+	-	SEN
-	+	-	+	-	SEN

Pulmonary	81
Extrapulmc	32

Samples		Extrapulmonary	
Pulmonary	82	CSF	13
CSF	13	PF	6
PF	6	Pus	6
Pus	6	SF	1
SF	1	UR	1
UR	1	BX	1
BX	1	LNA	3
LNA	3		

BAL	3
Sputum	76
GA	3

	Sen	FQ Res	XDR
Bactec MGIT-960	67	40	6
Genotype MTBDRsl V1.0	68	40	5
Genotype MTBDRsl V2.0	67	39	7

LPA v2
(n;%)
Sen (67;59.3)
FQ (39; 34.5)
XDR (7; 6.2%)

68
40
5
