# Supplementary material for: Performance of Genotype MTBDRsl V2.0 over the Genotype MTBDRsl V1 for detection of second line drug resistance: An Indian perspective
Source: PLoS One. 2020 Mar 4;15(3):e0229419. doi: 10.1371/journal.pone.0229419 (PMC7055869; doi:10.1371/journal.pone.0229419)
Supplement: Attachment — Submitted filename: Response_to_reviewers_PONE.docx [file pone.0229419.s002.docx]

|  | **Reviewers comments** | **Authors Reply** |
| --- | --- | --- |
| 1 | Please ensure that your manuscript meets PLOS ONE's style requirements, including those for file naming. | Thank you very much for the suggestion.  Manuscript has been modified as per PLOS ONE’s style requirements, including those file for naming. |
| 2 | Please provide additional details regarding participant consent. In the ethics statement in the Methods and online submission information, please ensure that you have specified (1) whether consent was informed and (2) what type you obtained (for instance, written or verbal, and if verbal, how it was documented and witnessed). If the need for consent was waived by the ethics committee, please include this information. | Consent was informed and written for samples received for routine TB investigations like smear microscopy, culture and DST Ethics reference number is provided (IHEC-LOP/2018/EF0102). |
| 3. | Please remove any funding-related text from the manuscript and let us know how you would like to update your Funding Statement. Currently, your Funding Statement reads as follows:  NO | Funding related text has been removed from the acknowledgement section and will be including in funding statement. |
| 4. | Please amend either the title on the online submission form (via Edit Submission) or the title in the manuscript so that they are identical | Title on online submission and manuscript has been amended accordingly. |
| 5. | It would be helpful to know what was the principle of specimens selection, and whether it essentially should be considered a convenience sample - this is necessary to put the results in the context, and decide if these could be considered generalizable/expandable to other areas in India. | The specimens selected in this study were convenience samples and included both pulmonary and extra-pulmonary samples received from different parts of India which were further tested for SIRE DST and second line DST (Rufai et al; Scientific Reports 2018). This study is an extension of the same study. The performance of genotype MTBDR*sl* V2.0 was evaluated over the Genotype MTBDR*sl* V1.0) on the aliquots of the same samples. The study has potential to be generalizable to other areas of India. |
| 6. | I strongly recommend that Table 3 is re-formatted and band patterns are reported as combined patterns with missing WT band and specific mutation bands visible; the way it reports patterns now is extremely confusing and misleading. | Table 3 as well as Table 2 have been formatted as per the suggestions. |
| 7. | I think expanding on cost issues would be really beneficial in the context of implementation of LPAs for rapid detection of XDR/pre-XDR isolates in India and similar settings. I was really surprised to learn cost of MTBDR*plus* version 2.0 was significantly higher compared to V1.0 which was not the case in any settings (including high prevalence) I am familiar with. More accurate information is needed on price per test (paper reports cost per kit and it's unclear if it per 12 test box or.?). I would encourage providing more information on prices and call for urgent action from international and more importantly national bodies and considerations on the role of private providers in TB laboratory and diagnostic management in India. | In India the first version (MTBDR*sl* V1.0) of LPA was available before the launch of Genotype MTBDR*sl* V2.0 costed ranging from (1389 to 1527) US dollar each kit for 96 tests, while the cost of new version (V2.0) is approximately (1666 to 1944) US dollar each kit of 96 tests (17.3 to 20.2) US dollar per test. The variation in rates are discounted rates based on if it is a government institutions, NGO (not for profit) or private sector. |
